# Toxicological Evaluation of Luminescent Silica Nanoparticles as New Drug Nanocarriers in Different Cancer Cell Lines

**DOI:** 10.3390/ma11081310

**Published:** 2018-07-28

**Authors:** Gonçalo Marcelo, Jessica Ariana-Machado, Maria Enea, Helena Carmo, Benito Rodríguez-González, José Luis Capelo, Carlos Lodeiro, Elisabete Oliveira

**Affiliations:** 1 BIOSCOPE Group, LAQV@REQUIMTE, Chemistry Department, Faculty of Science and Technology, University NOVA of Lisbon, Caparica Campus, 2829-516 Caparica, Portugal; g.marcelo@campus.fct.unl.pt (G.M.); ja.machado@campus.fct.unl.pt (J.A.-M.); jlcm@fct.unl.pt (J.L.C.); cle@fct.unl.pt (C.L.); 2 UCIBIO-REQUIMTE, Laboratory of Toxicology, Department of Biological Sciences, Faculty of Pharmacy of the University of Porto, 4050-313 Porto, Portugal; eneavmaria@gmail.com (M.E.); helenacarmo@ff.up.pt (H.C.); 3 Scientific and Technological Research Assistance Centre (CACTI), University of Vigo, 36310 Vigo, Spain; jbenito@uvigo.es; 4 PROTEOMASS Scientific Society, Rua dos Inventores, Madam Parque, Caparica Campus, 2829-516 Caparica, Portugal

**Keywords:** luminescence mesoporous silica nanoparticles, cytotoxicity, drug delivery

## Abstract

Luminescent mesoporous silica nanoparticles, CdTeQDs@MNs@PEG1, SiQDs@Isoc@MNs and SiQDs@Isoc@MNs@PEG2, were successfully synthetized and characterized by SEM, TEM, XRD, N_2_ nitrogen isotherms, ^1^H NMR, IR, absorption, and emission spectroscopy. Cytotoxic evaluation of these nanoparticles was performed in relevant in vitro cell models, such as human hepatoma HepG2, human brain endothelial (hCMEC/D3), and human epithelial colorectal adenocarcinoma (Caco-2) cell lines. None of the tested nanoparticles showed significant cytotoxicity in any of the three performed assays (MTT/NR/ LDH) compared with the respective solvent and/or coating controls, excepting for CdTeQDs@MNs@PEG1 nanoparticles, where significant toxicity was noticed in hCMEC/D3 cells. The results presented reveal that SiQDs-based mesoporous silica nanoparticles are promising nanoplatforms for cancer treatment, with a pH-responsive drug release profile and the ability to load 80% of doxorubicin.

## 1. Introduction

Mesoporous silica nanoparticles (MNs) are considered one of the most powerful mesoporous structures as they generally are bio-compatible and their surfaces can be modified, providing reservoirs for loading various functional molecules as nanocarriers and active sites for linking other targeted molecules by covalent association [1]. To facilitate the tracking of silica or silica-coated nanoparticles in a biological system, MNs are commonly labeled with fluorophores. Alternatively, due to their improved optical properties, quantum dots (QDs) have emerged as a new alternative luminescent source to fluorophores, avoiding time-consuming and expensive methods and several additional issues such as high toxicity [2,3,4]. As for QDs, their luminescence also provides an easy method for tracking nanocarriers into cells. Therefore, QDs functionalized with MNs may not only prevent QD agglomeration and resolve instability problems, but also offer a quantum confinement effect that benefits from the superior properties of both QDs and MNs [5]. The encapsulation of inorganic QDs with MNs has been widely investigated, and methods can be grouped into two general categories: The coating of inorganic QDs with MNs or the introduction of QDs into MNs pores (dye-doping).

Some papers have reported the successful preparation of silica-coated QDs. Wolcott et al. exploited the growth of an amorphous silica shell onto as-synthesized water-soluble CdTe QDs functionalized with thiols for bioconjugation to immunoglobulin-G-type proteins [6]. Song et al. further demonstrated the fabrication of mesoporous CdTe/ZnO@SiO_2_ core/shell nanostructures with tunable dual emission and ultrasensitive fluorescence response to metal ions [7]. Hu et al. synthesized a new generation of silica encapsulated single quantum dots [8]. Moreover, Zhou et al. fabricated mesoporous silica-coated CdTe QDs functionalized with folic acid for targeted lung cancer cell imaging [9].

The commonly used QDs are cadmium based, and their toxicity is still a problem in biological applications [10,11]. Silicon (Si) is a well-studied material in the semiconductor family, which has attracted great attention because of its intrinsic advantages, such as low cost, strong fluorescence, high stability, and photostability. Moreover, SiQDs have unique characteristics, such as low toxicity, favorable biocompatibility, and biodegradability, exhibiting significant potential in the biological field [12,13,14]. SiQDs are widely accepted as promising alternatives to toxic heavy-metal CdTe QDs. For SiQDs, different methods of synthesis have been reported, including ultrasonic dispersion of electrochemically etched silicon [15], laser-driven pyrolysis of silane [16], gas phase synthesis [17], and microemulsion synthesis [2]. However, for application in biological systems, SiQDs must be water-soluble. Based on this evidence, several methods for the aqueous synthesis of silicon quantum dots have been described in the literature [3,4,18,19,20]. Despite the intense efforts to construct numerous drug nanocarriers for tumor-target drug delivery, knowledge about the toxic, pharmacokinetic, and pharmacodynamic effects of these nanomaterials is still lacking [21].

Considering these previous statements, in this study, encapsulation of inorganic QDs (CdTe QDs, and SiQDs) was performed by coating QDs with MCM-41 type MNs, by the addition of MNs to a crude solution of inorganic QDs in an aqueous synthesis. This study is the first time that aqueous luminescent SiQDs have been covered with mesoporous silica nanoparticles. These nanocarriers were further functionalized with a polymer (polyethylene glycol) layer in order to increase the solubility and biocompatibility of these nanosystems in biological media, for in vitro and in vivo applications.

Regarding the potential biomedical applications of the newly synthesized nanocarriers, several experimental models were used to assess in vitro toxicological effects. These studies were performed with (i) the human hepatoma cell line HepG2 to predict potential hepatotoxicity, (ii) the human brain endothelial cell line hCMEC to predict the effect on the blood brain barrier after systemic administration, and (iii) the human Caco-2 cell line, which is a model of the intestinal epithelium, an expected target after oral exposure. As a proof-of-concept, DOX-loaded SiQDs@Isoc@MNs toxicity was evaluated using two cancerous cell lines including HepG2 and Caco-2 cells in order to predict their biological effect.

Encapsulation and release studies of different active targets, such as anticancer drugs (doxorubicin) and isolated proteins (bovine serum albumin (BSA), lysozyme (LYS), carbonic anhydrase (CA), ovalbumin (OVA), hemoglobin (Hb), myoglobin (Myb), and cytochrome c (CYT)) were also explored.

## 2. Materials and Methods

### 2.1. Chemicals

Thioglycolic acid (TGA, C_2_H_4_O_2_S, ≥99%), cadmium acetate dihydrate (Cd(CH_3_COO)_2_·2H_2_O, 99.5%), (3-aminopropyl)triethoxysilane (APTES, H_2_N(CH_2_)_3_Si(OC_2_H_5_)_3_, 99%), tetraethyl orthosilicate (TEOS, SiC_8_H_20_O_4_, 98%), ammonia (NH_3_, ≥99.9%), cetyltrimethylammonium bromide (CTAB, C_19_H_42_BrN, ≥98%), poly(ethylene glycol) BioUltra 3000, poly(ethylene glycol) BioUltra 6000, poly(ethylene glycol) BioUltra 8000, *p*-toluenesulfonyl chloride (CH_3_C_6_H_4_SO_2_Cl, ≥98%), hydrochloric acid (HCl, 37%), magnesium sulfate (MgSO_4_, ≥99.5%), sodium ascorbate, bovine serum albumin (BSA), lysozyme (LYS), carbonic anhydrase (CA), ovalbumin (OVA), hemoglobin (Hb), myoglobin (Myb), and cytochrome c (CYT) were purchased from Sigma-Aldrich (Lisbon, Portugal). Sodium hydroxide (NaOH, ≥98%) was produced by Panreac. Sodium tellurite (Na_2_TeO_3_, 99.5%) and ammonium nitrate (NH_4_NO_3_, 99.9%) were purchased from Alfa Aesar. Sodium borohydride (NaBH_4_, 99%), ethylene glycol (C_2_H_6_O_2_, ≥99.5%) and (3-isocyanatopropyl)triethoxysilane (IPTES, (C_2_H_5_O)_3_Si(CH_2_)_3_NCO, 95%) were produced by Fluka. Methanol (MeOH), ethanol (EtOH), tetrahydrofuran (THF, C_4_H_8_O, ≥99.9%), and chloroform (CHCl_3_) were purchased by Carlo Erba Reagents. All the reagents and solvents were of analytical reagent grade and were used as received. All cell culture reagents were purchased from Gibco (Alfagene, Lisbon, Portugal) unless stated otherwise.

### 2.2. Instrumentation

UV-Vis absorption spectra were acquired on a Jasco V-630 spectrophotometer (Jasco Corporation, Tokyo, Japan); nanoparticle size distributions and zeta potential were measured using a dynamic light scattering, a Malvern Nano ZS instrument with a 633 nm laser diode, from Proteomass–Bioscope facility (Caparica, Portugal). 

Transmission electron microscopy (TEM) images were obtained with a JEOL JEM 2010F operating (JEOL, Peabody, MA, USA) at 200 kV; TEM images were collected using a multiscan camera and Digital Micrograph software from Gatan.

Surface electron microscopy (SEM) was performed in a SEM-FIB—Zeiss Auriga CrossBeam (ZEISS, Jena, Germany), workstation at Laboratory of Nanofabrication, CENIMAT. Pore size distribution and surface area determination result from the measurement of N_2_ adsorption/desorption at 77 K in a Micromeritics ASAP 2010 (Micrometrics Instrument Corporation, Norcross, USA) (Accelerated Surface Area and Porosimetry), at the Laboratory of Analysis from FCT—UNL. Specific surface areas (S_BET_) were estimated using Brunauer Emmett and Teller (BET) method [22].

X-ray diffraction (XRD) patterns were performed with RIGAKU MiniFlex II X-ray diffractometer (Rigaku Corporation, Tokyo, Japan) equipped with a Cu-Kα source (30 kV/15 mA). Measurements were obtained for 2θ angles ranging from 2° to 45°.

Infrared spectra were recorded on a PerkinElmer BX (Perkin Elmer Inc., Beaconsfield, UK) or PerkinElmer Spectrum Two spectrometer (Perkin Elmer Inc., Llantrisant, UK). Proton nuclear magnetic resonance (^1^H NMR) spectra were recorded on a Bruker Avance (Bruker Biopsin AG, Fällanden, Switzerland) III 400 at FCT-University Nova of Lisbon, Portugal. The NMR spectrometers (Bruker Biopsin AG, Fällanden, Switzerland) are part of The National NMR Facility, supported by Fundação para a Ciência e a Tecnologia (RECI/BBB-BQB/0230/2012).

### 2.3. Synthesis of Polyethylene Glycol (PEG) Derivatives

#### Synthesis of PEG(3000)-Silane (PEG1) and TsO-PEG(8000)-Silane (PEG2)

PEG(3000)-Silane (PEG1) was synthesized based on the process reported in a previous study [23]. Briefly, a solution of 86.4 mg (2.16 mol) NaOH in 4 mL of bi-distilled water was prepared in a round-bottom flask. Then, 1.8 g (0.6 mmol) of the commercially available poly(ethylene glycol) BioUltra 3000 (PEG3000) in 10 mL THF were added to the previous solution. The resulting mixture was stirred for 1 h at 0 °C. Then, 0.137 g (0.72 mmol) of *p*-toluenesulfonyl chloride in 1 mL of THF were added dropwise to the reaction mixture over the course of 30 min at 0 °C. The mixture was stirred for an additional 3 h. Afterward, a solution of 1 M HCl (2 mL) was added. The organic phase was extracted three times with chloroform, dried over MgSO_4_, and filtered. The solvent was removed by rotary evaporation. The transparent crude product, showing the substitution of the terminal –OH group with –OTs, was used for the next step without further purification [23].

To the product, 0.140 mL (0.6 mmol) of APTES in 2.5 mL of chloroform were added and left at 70 °C for 8 h under reflux conditions to bind the silane group to the PEG-OT moiety through the amino functionality. The organic solvent was removed by rotary evaporation and the obtained raw product was stored at 4 °C [23].

**Scheme 1:**
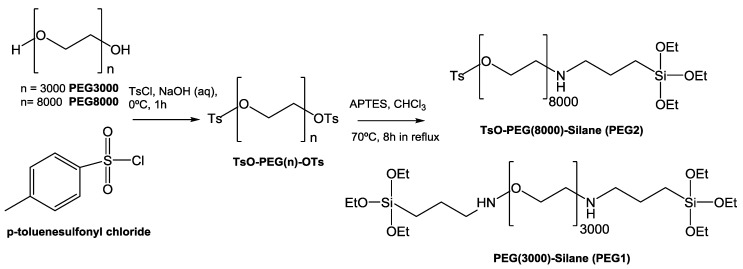
Synthetic pathway of PEG1 and PEG2.

For TsO-PEG(8000)-Silane (PEG2), the previous procedure was performed; however, a solution of 180 mg (4.5 mmol) NaOH in 8.33 mL of bi-distilled water was used. Then, 10 g (1.25 mmol) of commercially available poly(ethylene glycol) BioUltra 8000, 0.9342 g (4.9 mmol) of *p*-toluenesulfonyl chloride in 1 mL of THF and 130 μL (0.73 mmol, 1 eq) of APTMS in 2 mL chloroform were added to 6.061 g (0.73 mmol, 1eq) of TsO-PEG(8000)-OTs (Scheme 1). PEG1. ^1^H-NMR (CDCl_3_): 3.83 (t, Si(OCH_2_CH_3_), 3.71–3.58 (m, (OCH_2_CH_2_)_3000_), 3.46 (t, CH_2_CH_2_NH), 1.62 (m, CH_2_CH_2_Si), 1.27 (t, Si(OCH_2_CH_3_), 0.86 (m, CH_2_Si).

PEG2. ^1^H-NMR (CDCl_3_): 7.95 (d, ring CH, –SO_3_ side), 7.82 (d, ring CH, CH_3_– side), 4.37 (t, CH_2_CH_2_(OCH_2_CH_2_)_8000_), 4.18 (t, CH_2_(OCH_2_CH_2_)_8000_), 3.71–3.58 (m, (OCH_2_CH_2_)_8000_), 3.49 (q, (OCH_2_CH_2_)_8000_OCH_2_), 2.50 (d, CH_2_NH), 2.37 (m, NHCH_2_), 1.79 (s, CH_3_ tosyl), 1.65 (m, NHCH_2_CH_2_), 1.28 (m, Si(OCH_2_CH_3_), 0.90 (t, CH_2_Si).

In terms of infrared spectra, all PEG-derivatives showed the characteristic peaks at 2880 cm^−1^, 1466 cm^−1^, and 1097–1105 cm^−1^ related to –(CH_2_CH_2_)_n_ vibration and C-O ether bending vibration of the polymer. The bending vibration of the benzene ring at 748–752 cm^−1^ in PEG2 also indicates that PEG reacted with the TsCl.

### 2.4. Synthesis of Luminescent Mesoporous Silica Nanoparticles

#### 2.4.1. Synthesis and Functionalization of Cadmium Telluride Quantum Dots @ Mesoporous Silica Nanoparticles (CdTeQDs@MNs, CdTeQDs@MNs@PEG1)

CdTe QDs and CdTeQDs@MNs were synthetized as previously reported [24,25]. The main synthetic procedure is described below.

For CdTe QDs, Cd(CH_3_COO)_2_·2H_2_O (53 mg, 0.2 mmol) was dissolved in 50 mL of bi-distilled water in a stand-up flask (solution A). Then, 18 μL TGA was added, and the pH was adjusted to 10 by adding a solution of 1 M NaOH dropwise (by adding TGA, the solution becomes cloudy, and then the NaOH must be added until it is transparent). Afterward, 8.86 mg (0.04 mmol) of Na_2_TeO_3_, dissolved in 50 mL bi-distilled water (solution B), was added to the previous solution (solution A). Then, 80 mg of NaBH_4_ was added to the final mixture and stirred at room temperature for 5 min. After this, the reaction mixture was divided in half and transferred into two twin-neck round-bottom flasks, which were attached to a condenser and refluxed at ~200 °C under open-air conditions. The reaction time was controlled under UV light, resulting in green CdTe QDs after approximately 10 minutes [25].

CdTe QDs coated with MSNs (CdTeQDs@MNs). To the previous crude solution of CdTe QDs, 0.10 g of CTAB dissolved in 10 mL of bi-distilled water was added. This mixture was stirred for 30 min at 50 °C. In the following order, bi-distilled water (30 mL), ethylene glycol (10 mL), and NaOH 1M (165 µL) were added to the above mixture and stirred for a further 30 min at 70 °C. Then, 0.75 mL of TEOS were added dropwise to the mixture and left for 3 h at 70 °C under stirring. The final product was washed three times with a solution of bi-distilled water and methanol. For template removal, a solution of 60 mg of NH_4_NO_3_ in 20 mL of methanol was added to the previous washed product and transferred to a round-bottom flask, which was stirred for 30 min at 60 °C. The last three steps were repeated twice.

For the functionalization of CdTeQDs@MNs with PEG1, a total of 200 mg of PEG1 was dissolved in 6.5 mL bi-distilled water. Then, this solution was added to the green CdTeQDs@mSiO_2_ suspension with template (50 mg in 6.5 mL of bi-distilled water), followed by 3 h of reflux at 100 °C. The mixture was then stirred overnight at room temperature. The functionalized mesoporous nanoparticles were isolated by centrifugation (4000 rpm, 10 min), washed three times with bi-distilled water and twice with ethanol, and dried. The template was removed using the same procedure mentioned above. After, the final product was washed once with bi-distilled water and twice with methanol, and dried [26].

#### 2.4.2. Synthesis and Functionalization of Modified Luminescent Silicon Quantum Dots Coated with Mesoporous Silica Nanoparticles (SiQDs@Isoc@MNs, SiQDs@Isoc@MNs@PEG2)

SiQDs coated with (3-isocyanatopropyl)triethoxysilane (SiQDs@Isoc). The Si QDs were prepared by adding 1 mL of APTES to 4 mL of bi-distilled water while stirring. Then, 1.25 mL of 0.1 M of sodium ascorbate were added to the above mixture and stirred for 1 h at 40 °C [4,20]. Then, 2 mL of IPTES were added to the mixture and stirred for 2 h at room temperature. A white precipitate was formed after the addition of IPTES. The mixture containing the SiQDs@Isoc was centrifuged, washed three times with bi-distilled water, and dried.

SiQDs@Isoc coated with silica mesoporous nanoparticles (SiQDs@Isoc). A total of 200 mg of SiQDs@Isoc were added to 10 mL of an aqueous solution of 0.1 M CTAB. This mixture was stirred for 30 min at room temperature. Then, 500 µL of ammonia were added to the mixture above and stirred for a further 30 min at 50 °C, followed by the dropwise addition of 500 µL of TEOS. The final mixture was left for 2 h at 80 °C under stirring. The final product was centrifuged and washed three times with ethanol. For template removal, a solution of 60 mg of NH_4_NO_3_ in 20 mL of methanol was added to the previous washed product and transferred to a round-bottom flask which was stirred for 30 min at 60 °C. The last three steps were repeated twice. The reaction mixture was washed three times with bi-distilled water and methanol before being dried. The final mesoporous nanomaterials SiQDs@Isoc@MNs, was obtained as a powder.

For functionalization of SiQDs@Isoc@MNs with PEG2, a total of 200 mg of PEG2 were dissolved in 13 mL of bi-distilled water. Then, this solution was added to the SiQDs@Isoc@MNs suspension without a template (50 mg in 13 mL of bi-distilled water), followed by 3 h of reflux at 100 °C. The functionalized mesoporous nanoparticles were isolated by centrifugation, washed five times with bi-distilled water in Eppendorf tubes, and dried [26].

### 2.5. Toxicological Studies

#### 2.5.1. Cell Culture

Human colorectal adenocarcinoma (Caco-2) cell line and human liver hepatocellular (HepG2) cell line were supplied by the American Type Culture Collection (ATCC; Manassas, VA, USA) and routinely maintained in complete Dulbecco’s Modified Eagle’s Medium-high glucose (Sigma-Aldrich, Lisbon, Portugal) supplemented with 10% fetal bovine serum (FBS) and 1% penicillin-streptomycin. Cells were maintained in a humidified 5% CO_2_, 95% air atmosphere at 37 °C, and were passaged by trypsinization (0.25% trypsin/1 mM EDTA) when cells reached 70–80% confluence. The medium was changed every two days. The cells were subcultured over a maximum of 10 passages. For all studies, cells were seeded at a density 50,000 cells/well onto 96-well plates (BD Biosciences, Oxford, UK) in a volume of 100 μL of complete culture medium to obtain confluent monolayers within 24 h. Both cell lines were incubated with the nanoparticles in culture medium without FBS. The range of concentration was from 50 to 250 μg/mL for Caco-2 cells and from 150 to 250 μg/mL for HepG2 cells.

Immortalized hCMEC/D3 cells were obtained from the Institut National de la Santé et de la Recherche Médicale (INSERM, Paris, France) and routinely maintained in EndoGro-MV Basal medium (Milipore) (Sigma-Aldrich, Lisbon, Portugal) supplemented with 5% FBS, 1% antibiotic solution (10,000 U/mL penicillin, 10,000 µg/mL streptomycin), 0.2% EndoGro-LS Supplement, 5 ng/mL rh EGF, 10 mM l-glutamine, 1 µg/mL hydrocortisone hemisuccinate, 0.75 U/mL heparin sulfate, 50 µg/mL ascorbic acid, 5% FBS, 10 mM LiCl (99%, Calbiochem-Sigma-Merck, Lisbon, Portugal), 10 μM resveratrol (99%, Sigma-Aldrich, Lisbon, Portugal), and bFGF at 1 ng/mL (added extemporaneously). The cells were cultured on collagen-coated flasks at 37 °C in 95% humid air containing 5% CO_2,_ and the medium was changed every 2–3 days. The cells were passaged at approximately 80% confluence using a 0.25% trypsin/EDTA solution and used until passage number 35 in order to conserve their blood-brain barrier characteristics. For the assays, cells were seeded on collagen type I Cultrex (Enzifarma, Lisbon, Portugal)-pretreated 96-well plates with a density of 10,000 cells/well, and incubated for 4 days. After reaching confluence, the cells were incubated with the nanoparticles in complete culture medium at six distinct concentrations over a wide range starting from 50 μg/mL to 250 μg/mL.

To evaluate cytotoxicity, the MTT (3-(4,5-Dimethylthiazol-2-yl)-2,5-diphenyltetrazolium bromide) reduction assay and NR (neutral red) uptake were performed after 24 h of incubation for all three cell lines. The LDH leakage assay was additionally performed for HepG2 and Caco-2 cells.

For testing, stock solutions of 1 mg/mL of CdTeQDs@MNs@PEG1, SiQDs@Isoc@MNs@PEG2 and SiQDs@Isoc@MNs were prepared in 0.5 mM bovine serum albumin (BSA). Samples were kept at 4 °C, protected from light, and remained stable without any detectable sign of aggregation or precipitation throughout the study. The tested concentrations were diluted from the stock solution until just before use. Stock solutions were sonicated for 20 min in an ultrasound bath (Bandelin Sonorex RK 100H; Berlin, Germany) before dilution with the cell culture media. Stock solutions of 1 mg/mL of PEG1 and PEG2 in 0.5 mM BSA were also prepared and used as coating control, whereasz BSA was used as the solvent control. Two viability assays (MTT and NR) were used for testing doxorubicin-loaded SiQDs@Isoc@MNs (DOX-loaded SiQDs@Isoc@MN) in HepG2 and Caco-2 cell lines. The samples were dispersed using PBS pH 7.40 as in the in vitro release study and four final concentrations ranging from 25 to 250 μg/mL were tested for 2 and 24 h. A solvent control of PBS 5% pH 7.4 was also included.

#### 2.5.2. Cell Viability Assays

##### MTT Reduction Assay 

Metabolic activity was evaluated by the MTT reduction assay as previously described [27] with slight modifications. Briefly, the colorimetric assay is based on the reduction of the tetrazolium salt MTT (3-(4,5-Dimethylthiazol-2-yl)-2,5-diphenyltetrazolium bromide) to insoluble formazan crystals in the presence of mitochondrial enzymes. Only active enzymes present in viable cells have this ability, so the assay is considered a cell viability assay. The crystals are further dissolved in dimethyl sulfoxide (DMSO) and the absorbance is measured at 550 nm in a multi-well plate reader (BioTek Instruments, Winooski, VT, USA). Before the MTT assay, the cells were incubated with nanoparticles for 24 h at concentrations from 50 to 250 μg/mL for Caco-2 and hCMEC/D3 cells and 150 to 250 μg/mL for HepG2 cells. The media were collected for LDH quantification after the incubation period. The cells were further treated with 100 μL MTT solution to obtain a final concentration of 0.5 mg/mL for a specific time (3 h 30 min hCMEC/D3, 1 h 30 min Caco-2, and 20 min HepG2), and protected from light. For the solvent control, a final concentration of 0.125 mM BSA diluted in media was used. For the coating controls PEG1 and PEG2 solutions were diluted in media to obtain a final concentration of 0.25 mg/mL. Triton X-100 (1%; Sigma-Aldrich, Lisbon, Portugal) was used as positive control. Results are graphically presented as percentage of cell death relative to negative control. All concentrations were tested in 3 independent experiments run in triplicates.

##### NR Internalization Assay

The Neutral Red (NR) uptake assay is based on the ability of viable cells to incorporate and bind the neutral red dye inside lysosomes, so the amount of extracted dye is proportional to cell viability. Dead cells lose this ability to incorporate the dye. The assay was performed as previously described [28]. Briefly, after incubation with the nanoparticles for 24 h and removal of the cell culture media, the cells were incubated with 100 μL 50 μg/mL NR for a specific time (3 h 30 min hCMEC/D3, 1 h 30 min Caco-2, and 30 min HepG2) at 37 °C with 5% CO_2_, protected from light. Afterward, the excess NR solution was removed, and the dye was released from the lysosomes using a lysis solution (ethanol/glacial acetic acid). The absorbance was read at 540 nm and 690 nm using a multi-well plate reader (BioTek Instruments, Winooski, VT, USA). The same controls were used as those for the MTT assay, and the results are graphically presented as percentage of cell death relative to negative control. All concentrations were tested in at least three independent experiments run in triplicates.

##### Lactate Dehydrogenase (LDH) Release

LDH is a cytosolic enzyme released by the cell into the cell culture supernatant in case of damage of the plasma membrane. An increase in LDH release is proportional to the number of damaged cells. The method relies on the spectrophotometric measurement of the β-NADH cofactor consumed during the transformation of pyruvate to lactate in the presence of LDH. After removal of the cell culture media, prior to the MTT and NR assays, 10 µL of media from each test condition (nanoparticles, solvent control, coating control, and positive control) were transferred into another plate. KH_2_PO_4_ pH 7.4 buffer (40 µL of 0.05 M) and 200 µL of 0.15 mg/mL β-NADH cofactor were then added to each well, followed by the addition of 25 µL of 2.5 mg/mL sodium pyruvate immediately before the reading. The enzyme activity was observed at 340 nm wavelength using a multi-well plate reader (BioTek Instruments, Winooski, VT, USA). Results are graphically presented as LDH release to cell media compared to negative. All concentrations were tested in three independent experiments run in triplicates.

#### 2.5.3. Statistical Analysis

Statistical analysis was performed using GraphPad Prism 6 software (GraphPad Software, San Diego, CA, USA). The results of the MTT, NR, and LDH assays are presented as mean ± standard error of the mean (SEM) from three independent experiments run in triplicate. The normality of data distribution was assessed using the Kolmogorov-Smirnov, D’Agostino and Pearson, and Shapiro-Wilk tests. Statistical comparisons of groups were performed by one-way analysis of variance (ANOVA) followed by Tukey’s multiple comparison test when data followed a normal distribution. The Kruskal-Wallis test was followed by Dunn’s post hoc test otherwise. Solvent and coating controls were compared to the negative (cell culture media) control using the unpaired *t*-test. Significant differences were found relative to the negative control. Therefore, the test conditions were compared to the respective solvent control and/or coating controls. Significance was accepted at *p* values less than 0.05.

### 2.6. Protein Encapsulation Studies

Stock solutions (0.5 mg/mL) of BSA, lysozyme (LYS), carbonic anhydrase (CA), ovalbumin (OVA), hemoglobin (Hb), myoglobin (Myb), and cytochrome c (CYT) were used. A suspension of luminescent mesoporous nanoparticles (MNs) (2 mg/mL) in 1 mL of PBS was sonicated for 10 min. After that, 0.5 mL of proteins were mixed with 0.5 mL of luminescent nanoparticles. The final suspensions were stirred for 30 min. The samples were centrifuged, and the supernatant was quantified by absorption using the Bradford assay in a CLARIOstar^®^ High Performance Monochromator Multimode Microplate Reader (BMG LABTECH). Encapsulation efficiency (EE) and loading capacity were determined by the following equations where *t_protein_* is the total amount of protein/molecule and *f_protein_* is the amount of free protein/molecule) [29]:

(1) $$\mathrm{EE}\;\left(\%\right)=\;\frac{t_{protein-\;f_{protein}}}{t_{protein}}\times 100\%$$

(2) $$\mathrm{loading}\;\mathrm{capacity}\;\left(\mathrm{mg}/g\right)=\;\frac{t_{protein\;\left(\mathrm{mg}\right)-\;f_{protein\;\left(\mathrm{mg}\right)}}}{\mathrm{amount}\;\mathrm{of}\;\mathrm{nanoparticles}}$$

This study was performed with luminescent nanoparticles, CdTeQDs@MNS@PEG1, SiQDs@Isoc@MNs, and SiQDs@Isoc@MNs@PEG2.

### 2.7. Doxorubicin Loading and In Vitro Release Studies

As a proof-of-concept, the loading capacities of SiQDs@Isoc@MNs were tested with doxorubicin as the main testing drug. Briefly, 5 mg of the nanoparticle’s powder were first dissolved, under continuous ultrasonication for 5 min in 1.95 mL of a PBS pH 7.4 buffer solution. Subsequently, 50 µL of a doxorubicin stock solution (5 mg/mL) were added. The resulting mixture was left under continuous stirring for 24 h at room temperature. Pellets were recovered by centrifugation and supernatants quantified by UV.

Release profiles were traced in two different saline PBS buffer solutions of pH 5.4 and pH 7.4. Each pellet was resuspended in the corresponding solution and left under stirring for up to 72 h at 37 °C. Aliquots of the supernatant were collected every 10 min for the first 2 h, and then hourly for the next 5 h of incubation. The next collection points occurred at 24 h and 72 h. All supernatants were analyzed after centrifugation by UV.

## 3. Results and Discussion

### 3.1. Synthesis and Characterization of Luminescence Mesoporous Silica Nanoparticles

Different luminescent mesoporous silica nanoparticles, CdTeQDs@MNs, CdTeQDs@MNs@PEG1, SiQDs@Isoc@MNs, and SiQDs@Isoc@MNs@PEG2, were synthesized and their cytotoxicity evaluated in relevant in vitro cell models, such as human hepatoma HepG2, human brain endothelial (hCMEC), and human epithelial colorectal adenocarcinoma (Caco-2) cell lines. Moreover, the ability of these nanocarriers to load proteins and small molecules, such as doxorubicin, are also discussed.

In order to improve the stability and biocompatibility of CdTeQDs@MNs nanoparticles, already published by us in [24], a polymer PEG1 was functionalized onto mesoporous silica surface, leading to CdTeQDs@MNs@PEG1 nanoparticles.

After removing the surfactant of the nanoparticles, the obtained powder was characterized by solid-state emission spectra. We observed a maximum emission at 567 nm, corresponding to CdTeQDs@MNs@PEG1 nanoparticles.

**Figure 1:**
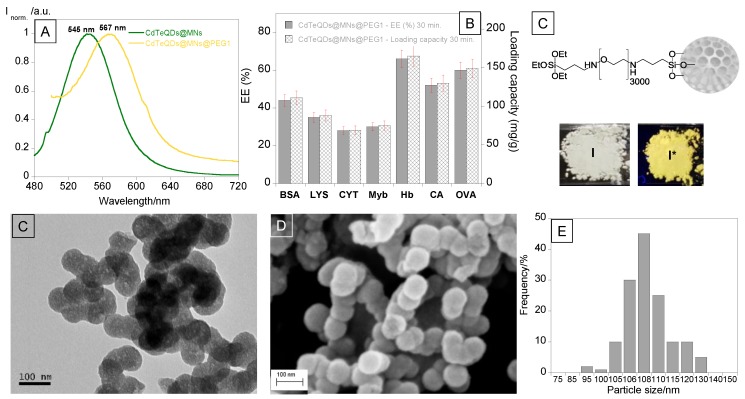
(**A**) Solid-state emission spectra of green CdTeQDs@MNs and CdTeQDs@MNs@PEG1 nanoparticles. (**B**) Encapsulation efficiency (EE%) and corresponding loading capacity (mg/g) (**B**) of seven model proteins in CdTeQDs@MNs@PEG1 for an initial weight ratio between protein and particles of 1:4 (250 mg of protein/1 g particles). (**C**) Schematic representation, (**I**) naked-eye and (I*) under ultraviolet (UV) lamp images of CdTeQDs@MNs@PEG1 nanoparticles. TEM (**D**), SEM (**E**) images and size histogram (**F**) of CdTeQDs@MNs@PEG1 nanoparticles.

Despite that no significant reduction in the fluorescence was observed, a red shift of 26 nm (from 545 nm to 567 nm) was visualized in the maximum of the emission band when compared to green CdTeQDs@MNs [24]. As a result, the powder’s color changed under UV irradiation from green to yellow, as seen in Figure 1.

Infrared spectra of the CdTeQDs@MNs@PEG1 nanoparticles were obtained to confirm the chemical binding of PEG1. PEGylated nanoparticles showed a characteristic peak of C–H at 2980 cm^−1^ corresponded to C–H from methyl and methylene groups of the polymer chain. Additionally, peaks at 1366 cm^−1^ and 1206 cm^−1^ are characteristic of NH and CH of the polymer backbone, respectively.

Dynamic light scattering revealed that both nanoparticles were negatively charged. However, the introduction of a PEG polymer led to the increase in nanoparticle stability, resulting in a more negative and stable zeta potential (changes from ca. −13.4 ± 0.2 mV to −34.3 ± 0.7 mV). As such, the aggregation state decreased, as can be seen by the decrease in PDI value from 0.4 to 0.2 [24]

The morphology of CdTeQDs@MNs@PEG1 nanoparticles was confirmed by TEM and SEM images, which confirmed the spherical shape of the nanoparticles. Through analysis of the TEM and SEM images, we noticed that CdTeQDs@MNs and CdTeQDs@MNs@PEG1 nanoparticles have an average size of ca. 105 ± 8 nm and 108 ± 6 nm, respectively.

Silicon quantum dots (SiQDs) were synthesized following the method reported by Wang et al. [4], with some modifications. Briefly, SiQDs were obtained in a one-step synthesis in water and open-air conditions as described in the experimental section. APTES was used as the silica source and sodium ascorbate as the reducing agent. Green fluorescent SiQDs were obtained and characterized by dynamic light scattering, fluorescence quantum yield, as well as absorption and emission spectroscopy. Thus, SiQDs presented a size of 3.6 nm and a fluorescence quantum yield of ca. 13%.

**Figure 2:**
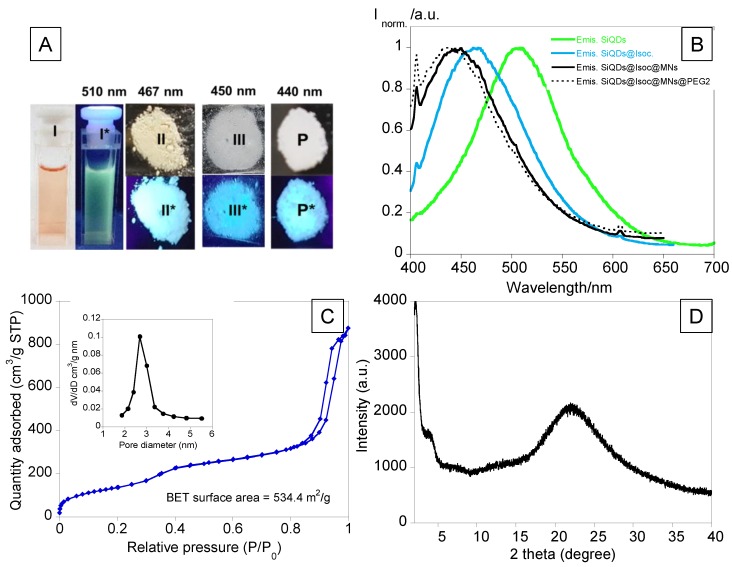
(**A**) Naked-eye images (I, II, III, P) and under an emissive UV lamp (λ_exc_=365 nm) (I*, II*, III*, P*) of SiQDs (I, I*), SiQDs@Isoc (II, II*), SiQDs@Isoc@MNs (III, III*), SiQDs@Isoc@MNs@PEG2 (P, P*). (**B**) Emission spectra of SiQDs and Solid-state emission spectra of SiQDs@Isoc, SiQDs@Isoc@MNs and SiQDs@Isoc@MNs@PEG2. (**C**) Nitrogen physio sorption isotherms and pore size distribution (inset). (**D**) X-ray diffraction (XRD) of SiQDs@Isoc@MNs.

Regarding the absorption spectra, two bands, one at 370 nm and another at ca. 520 nm, characteristic of its red naked-eye color, were observed. As for the emission spectra, SiQDs showed a band with a maximum at 510 nm (Figure 2).

**Scheme 2:**
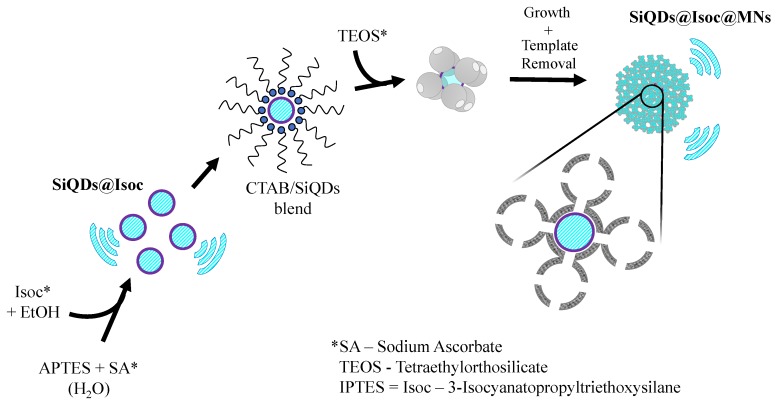
Schematic synthetic pathway of SiQDs@Isoc@MNs.

The zeta potential was negative, with a value ca. –4.1 ± 0.5 mV, revealing the instability of these quantum dots. To overcome this issue and increase stability, the crude SiQDs were coated with a layer of IPTES (SiQDs@Isoc). IPTES was added to the fluorescent SiQDs to create a centered core and avoiding further delivery of the QDs through the pore. SiQDs@Isoc was obtained as a white powder, with an emissive blue color with a maximum band centered at ca. 467 nm (Figure 2). After that, a layer of MSNs was added, as can be seen in the experimental section (Scheme 2).

The resulting nanoparticles SiQDs@Isoc@MNs were white with an emission band centered at ca. 450 nm and zeta potential of ca. 30.0 ± 1.2 mV (Figure 2).

Aiming to improve its solubility and reduce aggregation, SiQDs@Isoc@MNs were functionalized with the polymer PEG2, and as a result, improvements in the PDI (from ca. 0.9 to 0.5) and zeta potential (from ca. 30.0 ± 1.2 mV to 34.8 ± 2.8 mV) were observed. Additionally, a blue shift from 450 nm to 440 nm in the emission spectra after PEG functionalization indicated the surface coating of nanoparticles (Figure 2).

All synthetic steps were followed by infrared spectroscopy (IR). The typical peaks at 1070 and 692 cm^−1^, characteristic of the silica framework (Si–O–Si) and of the Si-C stretching vibration, were detected, respectively. Moreover, the vibration at ca. 1635 cm^−1^ related to the bending modes of physiosorbed water was also observed.

The different layers were confirmed by IR, in which the peak at 1740 cm^−1^ confirms the layer of isocyanate (C=O), and the peak at ca. 1565 cm^−1^ of NH vibration confirms the functionalization of the PEG polymer. The vibration –(CH_2_CH_2_)_n_ of the polymer was observed at ca. 2974–2874 cm^−1^ and ca. 1443 cm^−1^, respectively. Moreover, an additional absorption peak at ca. 1200 cm^−1^ was attributed to the C–N stretching vibration of the polymer.

In order to extend our knowledge about the structural and morphological aspects of the synthetized SiQDs@Isoc@MNs, additional analyses including X-ray diffraction (XRD), nitrogen physio sorption isotherms, scanning electron microscopy (SEM), and transmission electron microscopy, were performed.

Figure 2C shows nitrogen adsorption-desorption type IV isotherms of ordered mesoporous. The BET surface area and pore diameters were 534 m^2^/g and 2.8 ± 0.3 nm, respectively. The XRD spectra of the SiQDs@Isoc@MNs sample (Figure 2D) were in accordance with the most reported amorphous silicas, with the characteristic SiO_2_ broad band and its equivalent Bragg angle at 22° present [30].

**Figure 3:**
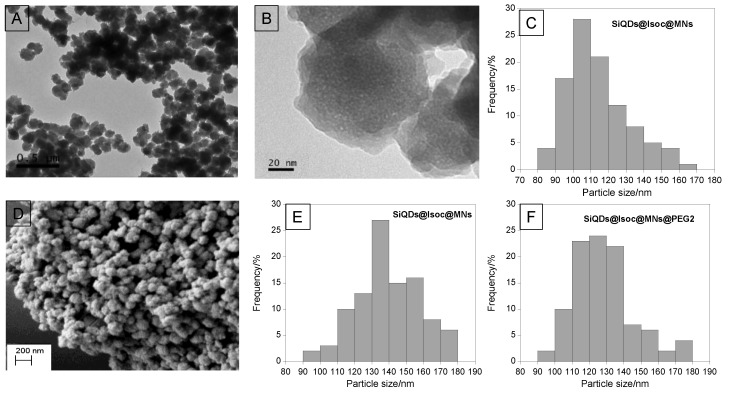
TEM (**A**,**B**) and SEM (**D**) images of SiQDs@Isoc@MNs nanoparticles. Size distribution histograms of TEM (**C**) and SEM (**E**) images of SiQDs@Isoc@MNs nanoparticles. (**F**) Size distribution histogram of SEM image of SiQDs@Isoc@MNs@PEG2 nanoparticles.

The TEM and SEM images proved the spherical shape of the nanoparticles, as well as their pore structure (Figure 3B), represented by the white dots. Through TEM and SEM images, we noticed that SiQDs@Isoc@MNs nanoparticles have a size around 114 ± 17 nm and ca. 139 ± 18 nm, respectively (Figure 3C,E). Moreover, through the SEM images, we observed a size decrease in the nanoparticles after functionalization with PEG2, from 139 ± 18 nm to 128 ± 11 nm (Figure 3E,F).

### 3.2. In Vitro Toxicological Studies to Characterize the Toxicity Profile and Respective Mechanisms of the Different Nanomaterials

**Figure 4:**
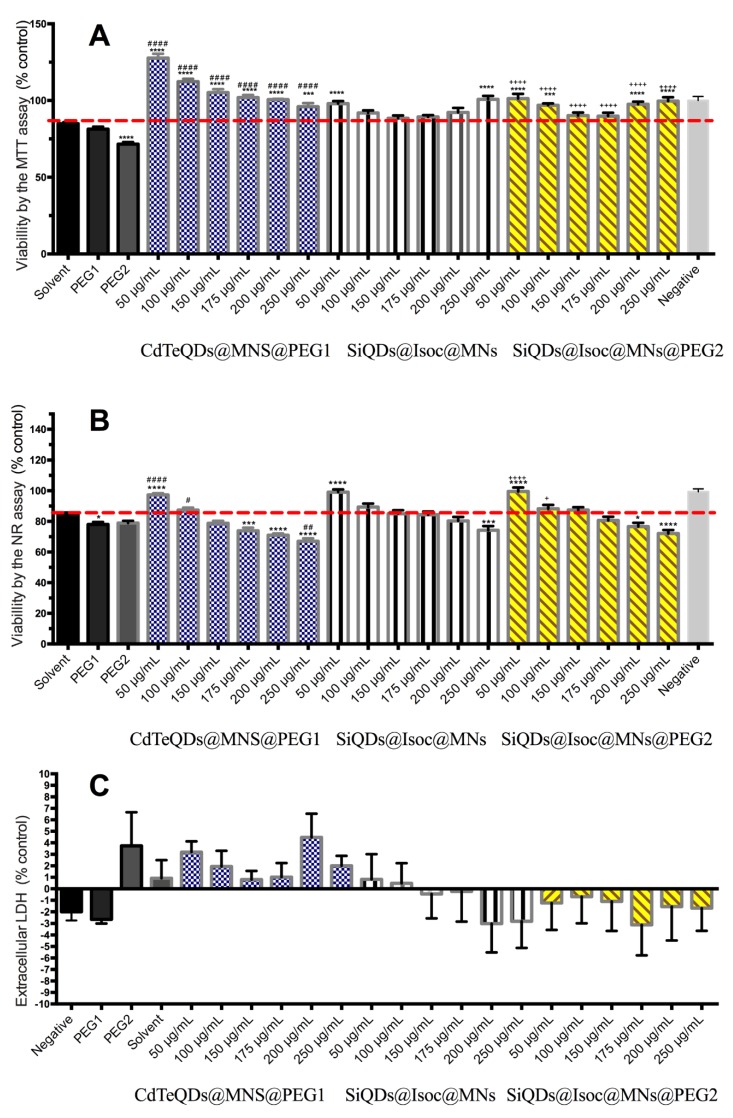
MTT reduction assay (**A**), NR incorporation assay (**B**) and LDH release (**C**) after 24 h incubation of CdTeQDs@MNs@PEG1, SiQDs@Isoc@MNs, and SiQDs@Isoc@MNs@PEG2 (50–250 μg/mL) for Caco-2 cell line. Results are expressed as mean ± SEM (*n* = 3 independent experiments). Statistical comparisons were performed using one-way ANOVA/Tukey’s multiple comparison test (* *p* < 0.05; *** *p*< 0.001; **** *p* < 0.0001 vs. BSA 0.125 mM solvent control; ^#^ *p* < 0.05; ^##^ *p* < 0.01; ^####^ *p* < 0.0001 vs. PEG1 coating control; ^+^ *p* < 0.05;; ^++++^ *p* < 0.0001 vs. PEG2 coating control).

Caco-2 cells proved to be the most resistant of all the tested cell lines. None of the tested nanoparticles showed significant cytotoxicity in any of the three performed assays (MTT, NR, or LDH) compared with the respective solvent and/or coating controls, suggesting that any of the three tested nanoparticles can be used for oral administration (Figure 4).

The coatings (both PEG1 and PEG2) produced toxicity when compared with the negative control in the MTT and NR assay, but it seems that the treatment of the NPs with the coating reduced the toxic effect of the coating itself (PEG1 and PEG2) when the metabolic function was evaluated by the MTT assay (*p* < 0.01). This protective effect was, however, not noted in the NR assay. The low toxicity of PEG-coated silica nanoparticles in Caco-2 cells has already been reported [31], as well as for mesoporous silica nanoparticles used for increasing the oral bioavailability and permeation for poorly water-soluble drugs [32]. Nevertheless, in our study, even if the NR assay showed a slight decrease in cellular viability for higher concentrations of CdTeQDs@MNs@PEG1 and SiQDs@Isoc@MNs@PEG2, these differences were not statistically significant when compared with the respective coating control and with the non-coated SiQDs@Isoc@MNs. In the case of SiQDs@Isoc@MNs, the viability decrease was statistically significant but without an expected biological significance compared to controls (below 20%).

**Figure 5:**
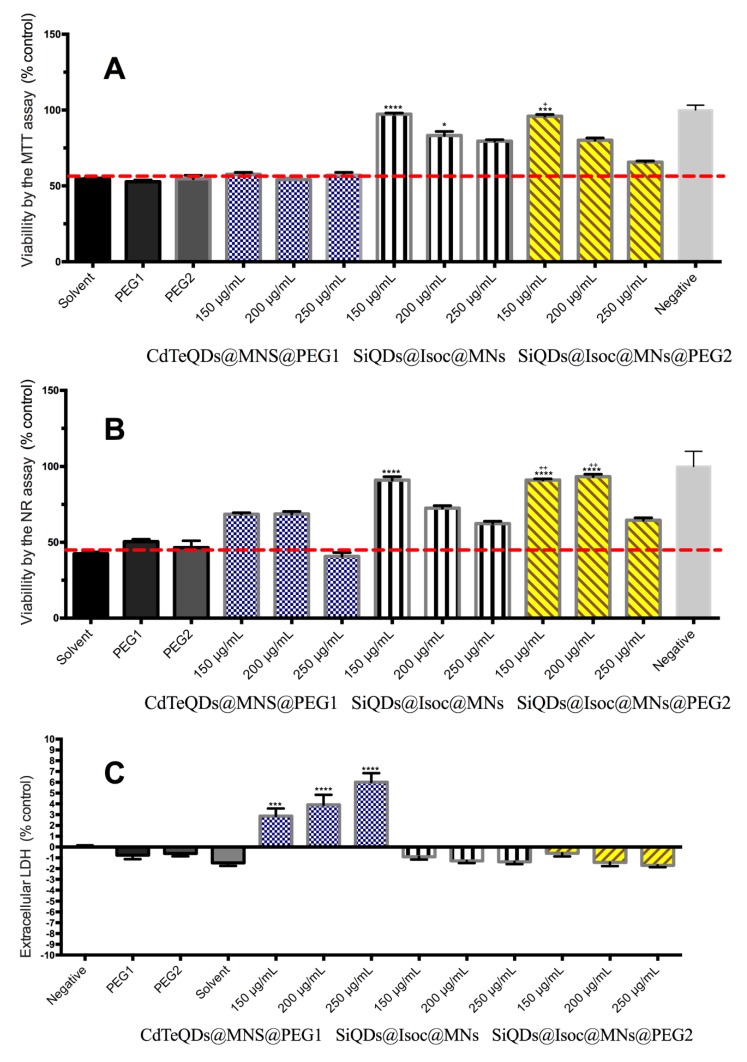
MTT reduction assay (**A**), NR incorporation assay (**B**) and LDH release (**C**) after 24 h incubations of CdTeQDs@MNs@PEG1, SiQDs@Isoc@MNs and SiQDs@Isoc@MNs@PEG2 (150 μg/mL–250 μg/mL) for HepG2 cell line. Results are expressed as mean ± SEM (*n* = 3 independent experiments). Statistical comparisons were completed using one-way ANOVA/Dunn’s multiple comparison test (* *p* < 0.05; *** *p* < 0.001; **** *p* < 0.0001 vs. BSA 0.125 mM solvent control; vs. PEG1 coating control; ^+^ *p* < 0.05; ^++^ *p* < 0.01; vs. PEG2 coating control).

None of the nanoparticles produced toxic effects on HepG2 cells in any of the assays (MTT, NR, and LDH) when compared with the solvent control (Figure 5). The lack of a toxic effect of PEG-coated mesoporous silica nanoparticles was already reported for this hepatic cell line [33]. While CdTe quantum dots alone present toxicity on HepG2 cells [34] after 6, 12, and 24 h incubations, at concentrations up to 1 μg/mL in Cd^2+^ (with mitochondrial swelling and mitochondrial membrane disruption, increase in Ca^2+^ intracellular levels, decrease in cellular respiration and depression of ATP levels), the silica coating of these CdTe quantum dots is expected to reduce this toxicity [35]. The presence of a polymer coating like CdTeQDs@MNs@PEG1 is already known to influence their toxicological profile and reduce the release of toxic Cd^2+^ [31]. Our data indicate a predictably low hepatotoxic effect of these nanomaterials.

**Figure 6:**
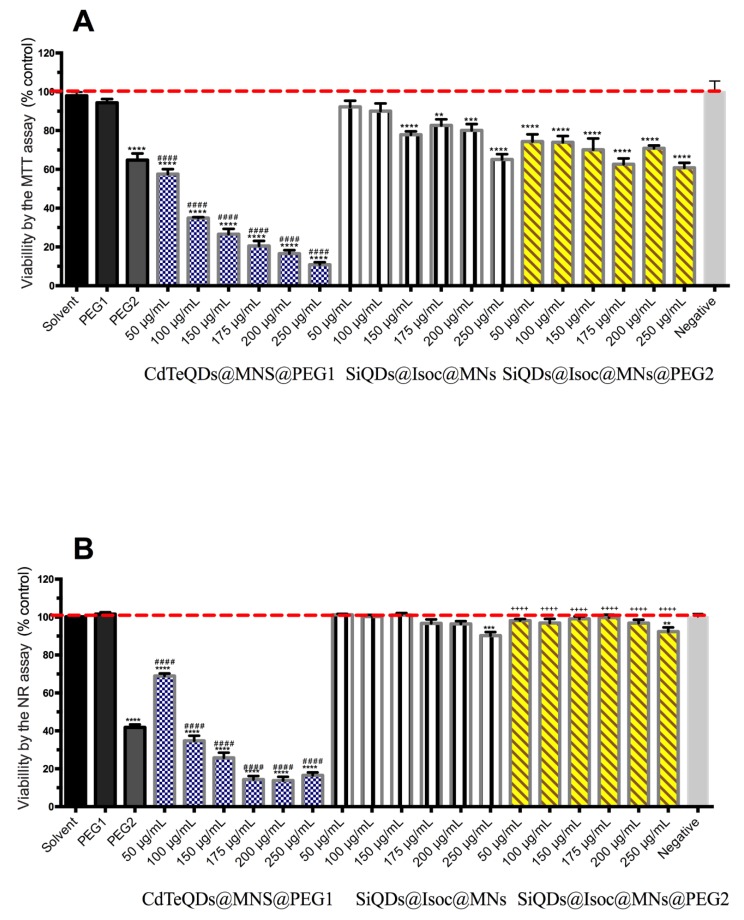
MTT reduction assay (**A**) and NR incorporation assay (**B**) after 24 h incubations of CdTeQDs@MNs@PEG1, SiQDs@Isoc@MNs and SiQDs@Isoc@MNs@PEG2 (50 μg/mL–250 μg/mL) for hCMEC/D3 cell line. Results are expressed as mean ± SEM (*n* = 3 independent experiments). Statistical comparisons were made using one-way ANOVA/Tukey’s multiple comparison test (** *p* < 0.01; *** *p* < 0.001; **** *p* < 0.0001 vs. BSA 0.125 mM solvent control; ^####^ *p* < 0.0001 vs. PEG1 coating control; ^++++^ *p* < 0.0001 vs. PEG2 coating control).

Human endothelial cells (hCMEC/D3) were the most sensitive to the effect of the nanoparticles. In the PEG2 coating control, a reduction of viable cells when compared with the solvent control for both MTT and NR assays (*p* < 0.0001) was observed (Figure 6). This cytotoxicity induced by the SiQDs@Isoc@MNs@PEG2 as determined in the MTT assay, was not confirmed by the NR assay, since no effect was observed for any of the PEG2-coated nanoparticles, despite obtaining the same cytotoxicity results for the coating control. Therefore, the nanoparticles appear to offer some protection against the coating effect, at least at the lysosomal level (*p* < 0.0001). The same type of NPs but without capping, the SiQDs@Isoc@MNs, showed a toxic effect in mitochondria but with biological significance only at the highest concentration (*p* < 0.0001). CdTeQDs@MNs@PEG1 presented concentration-dependent toxicity in both assays, with a reduction in viability until 16.54%, thus proving to be the most cytotoxic among the tested nanoparticles (*p* < 0.05). The main reason for this toxic effect is attributed to the release of toxic Cd^2+^ ions from the particles and the precipitation of particles in the cells [36,37]. With our data, we can anticipate a potentially detrimental effect for the systemic administration of these CdTeQDs@MNs@PEG1 at the BBB.

**Figure 7:**
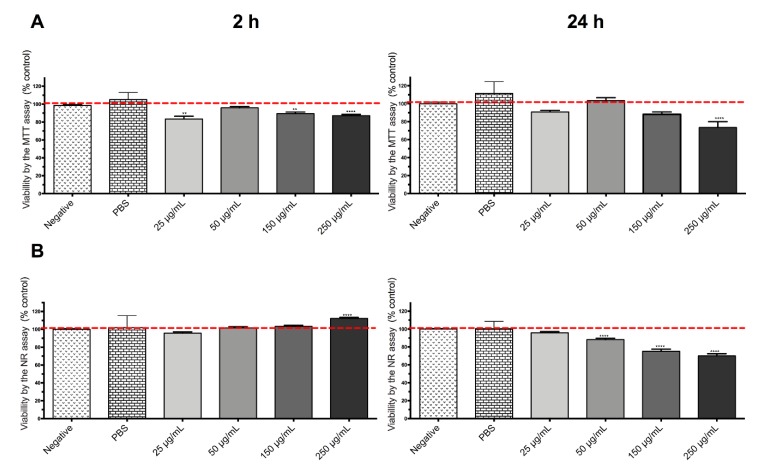
MTT reduction assay (**A**) and NR incorporation assay (**B**) after 4 and 24 h incubations of DOX-loaded SiQDs@Isoc@MNs (25 μg/mL–250 μg/mL) for Caco-2 cell line. Results are expressed as mean ± SEM (*n* = 3 independent experiments). Data are presented as percentage of cell death relative to the respective negative control. Statistical comparisons were made using one-way ANOVA/Dunn’s multiple comparison test (** *p* < 0.01; **** *p* < 0.0001 vs. negative control).

**Figure 8:**
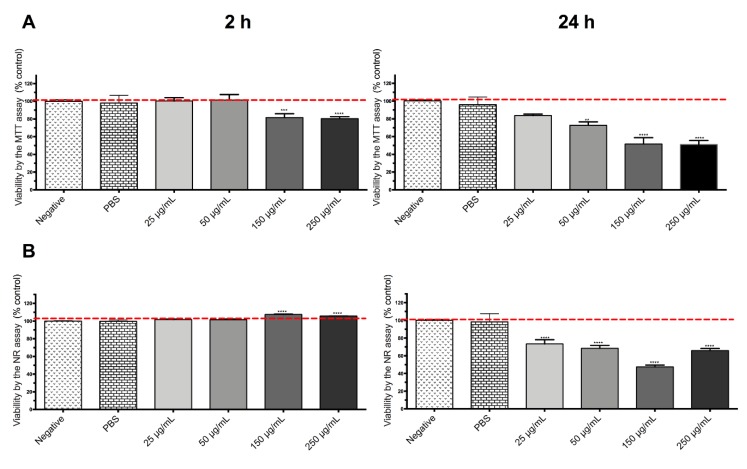
MTT reduction assay (**A**) and NR incorporation (**B**) after 4 and 24 h incubations of DOX-loaded SiQDs@Isoc@MNs (50 μg/mL–150 μg/mL) for HepG2 cell line. Results are expressed as mean ± SEM (*n* = 3 independent experiments). Data are presented as percentage of cell death relative to the respective negative control. Statistical comparisons were made using one-way ANOVA/Dunn’s multiple comparison test (** *p* < 0.01; *** *p* < 0.001; **** *p* < 0.0001 vs. negative control).

DOX-loaded SiQDs@Isoc@MNs were toxic for both cell lines. HepG2 proved to be more sensitive than Caco-2 with a maximum of toxicity at 24 h (Figure 7 and Figure 8). No biologically significant toxicity was noticed for Caco-2 after 2 h in either the MTT or NR assays, whereas after 24 h, viability decreased at the two highest concentrations, 150 and 250 μg/mL (*p* < 0.0001).

No biologically significant toxicity was noticed for HepG2 after 2 h (Figure 8). However, a toxic effect was noticed in HepG2 cells after 24 h, which was much more pronounced than for Caco-2 cells, with a decrease in viability up to 30% (MTT) and 47% (NR) at the highest tested concentrations (*p* < 0.0001). For both cell lines, toxicity appeared to be time-dependent. Considering that SiQDs@Isoc@MNs itself did not produce a toxic effect in either Caco-2 or HepG2 cells, the toxicity presented by the doxorubicin loaded SiQDs@Isoc@MNs is most likely due to the release of DOX from the nanoparticles during the incubation time.

### 3.3. Encapsulation and Release Studies

Encapsulation studies of the CdTeQDs@MNS@PEG1, SiQDs@Isoc@MNs and SiQDs@Isoc@MNs@PEG2 mesoporous nanoparticles in physiological pH (PBS, pH = 7.4) with single proteins exhibiting different sizes and isoelectric point (pI), such as BSA, LYS, CYT, Myb, Hb, CA and OVA. The proteins were incubated with the nanoparticles in PBS, with a weight ratio of 1:4 (protein:MSNs) for an incubation time of 30 min. Percentage of encapsulation (%EE) and the amount adsorbed were determined according to the experimental section.

Zeta potential values revealed a negative surface charge for CdTeQDs@MNs@PEG1 (−34.3 ± 0.7 mV) and a positive charge for SiQDs@Isoc@MNs (30.0 ± 1.2 mV) and SiQDs@Isoc@MNs@PEG2 (34.8 ± 2.8 mV).

Based on charge effect, a more efficient encapsulation for proteins with positive surface charge (LYS, CYT) in CdTeQDs@MNs@PEG1 and negative surface charge (BSA, CA and OVA) should be expected in the case of SiQDs@Isoc@MNs and SiQDs@Isoc@MNs@PEG2. Generally, no significant selective behavior was observed, and similar encapsulation values and loading capacities were obtained for all proteins. In CdTeQDs@MNs@PEG1, we obtained an %EE of around 40% for all proteins, except for Hb, CA, and OVA, with %EE values around 70% and a loading capacity of ca. 150–200 mg/g, indicating that electrostatic interactions were not the main driving force for protein encapsulation.

Concerning SiQDs@Isoc@MNs and SiQDs@Isoc@MNs@PEG2 nanoparticles, no significant affinity changes were observed in all proteins, with %EE values around 50% and loading capacities of ca. 100–120 mg/g. Moreover, the addition of PEG slightly decreased the affinity of the nanoparticles to the proteins.

Considering the initial weight ratio between the proteins and MSNs (1:4) and the %EE, these results showed that these nanoparticles could act as nanocarriers to efficiently encapsulate a wide variety of proteins.

### 3.4. Doxorubicin Loading and Release In Vitro Studies

Considering the obtained toxicological studies, the less toxic nanoparticles, SiQDs@Isoc@MNs, were tested as a nanocarriers with an anticancer model drug. The SiQDs@Isoc@MNs were incubated with a known amount of doxorubicin (DOX) in PBS pH 7.4, and the percentage of encapsulation and loading capacity were determined by UV technique, as described in the experimental section. The obtained DOX values for EE% and loaded amount were of 80% and 40 mg/g, respectively.

**Figure 9:**
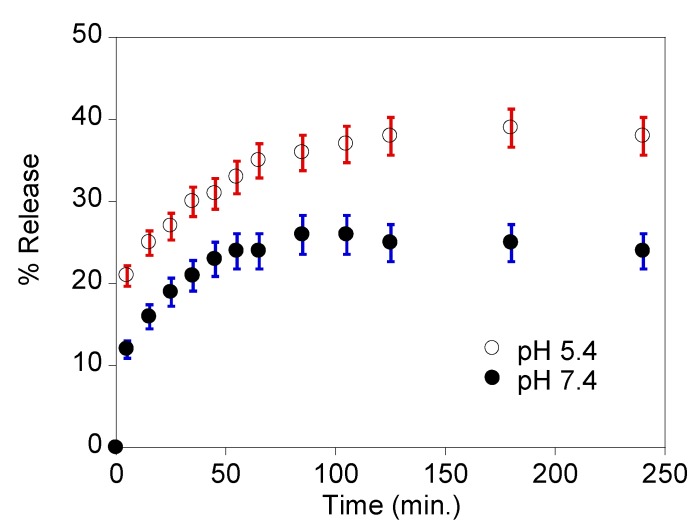
Percentage release profile of doxorubicin from the nanocarrier SiQDs@Isoc@MNs at pH 5.4 and pH 7.4.

In vitro release profiles were also obtained in two PBS solutions of pH 5.4 and 7.4, where drug release was favored at pH 5.4, with release percentages of ca. 40% and 20% in 60 min, respectively (Figure 9). Despite the absence of a total release of DOX from our nanocarrier, these values are quite similar to the ones obtained by Tang et al. [38], where a Magnetic Mesoporous Silica nanoplatform had a cumulative DOX release of ca. of 45% and 20% at pH 6.0 and pH 7.4 at 60 min, respectively. However, as no release control-coating was added to our nanocarriers, we concluded that our positively charged nanocarrier SiQDs@Isoc@MNs is pH-responsive, making it a good nanoplatform for cancer treatment due to this synergistic effect.

## 4. Conclusions

Different luminescent inorganic quantum dots (CdTeQDs and SiQDs) were successfully covered with mesoporous silica nanoparticles (MCM-41 type) and further functionalized with polyethylene glycol derivatives, resulting in CdTeQDs@MNs@PEG1, SiQDs@Isoc@MNs, and SiQDs@Isoc@MNs@PEG2 nanocomposites. Characterization techniques revealed the spherical shape of all nanoparticles, with an average size of 108 ± 6 nm (TEM) for CdTeQDs@MNs@PEG1, 114 ± 17 nm (TEM), 139 ± 18 nm (SEM) for SiQDs@Isoc@MNs, and 128 ± 11 nm (SEM) for SiQDs@Isoc@MNs@PEG2 nanoparticles. The addition of distinct layers was confirmed by infrared spectroscopy. The families of nanoparticles showed different zeta potential values; CdTeQDs@MNs@PEG1 was negatively charged, and SiQDs@Isoc@MNs and SiQDs@Isoc@MNs@PEG2 were positively charged. Despite the different charges, no significant selective affinity results were observed for any of the proteins studied. Regarding their cytotoxicity evaluation, Caco-2 cells proved to be the most resistant of all tested cell lines, without any significant toxicity in the three performed assays (MTT, NR, or LDH). Similar results were observed in HepG2 cells, showing the low hepatotoxic effect of these nanoparticles. However, CdTeQDs@MNs@PEG1 nanoparticles presented concentration-dependent toxicity in hCMEC/D3 cells, proving to be the most cytotoxic among the nanoparticles. Additionally, SiQDs@Isoc@MNs revealed good loading values for doxorubicin (80%). Based on in vitro release profiles, this positively charged nanocarrier is pH-responsive, so is thus a promising nanoplatform for cancer treatment.
